# Optimized technique for speaker changes detection in multispeaker audio recording using pyknogram and efficient distance metric

**DOI:** 10.1371/journal.pone.0314073

**Published:** 2024-11-20

**Authors:** Sukhvinder Kaur, Chander Prabha, Ravinder Pal Singh, Deepali Gupta, Sapna Juneja, Punit Gupta, Ali Nauman

**Affiliations:** 1 Swami Devi Dyal Institute of Engineering and Technology, Panchkula, Haryana, India; 2 Chitkara University Institute of Engineering and Technology, Chitkara University, Punjab, India; 3 Thapar Institute of Engineering and Technology, Punjab, India; 4 Department of Computer Science and Engineering (AI), KIET Group of Institutions, Ghaziabad, India; 5 School of Computer Science, University College Dublin, Dublin, Ireland; 6 Department of Computer Science and Engineering, Pandit Deendayal Energy University, Gandhinagar, India; 7 Department of Computer Science and Engineering, Yeungnam University, Gyeongsan, Republic of Korea; National Institute of Technology Uttarakhand, INDIA

## Abstract

Segmentation process is very popular in Speech recognition, word count, speaker indexing and speaker diarization process. This paper describes the speaker segmentation system which detects the speaker change point in an audio recording of multi speakers with the help of feature extraction and proposed distance metric algorithms. In this new approach, pre-processing of audio stream includes noise reduction, speech compression by using discrete wavelet transform (Daubechies wavelet ‘db40’ at level 2) and framing. It is followed by two feature extraction algorithms pyknogram and nonlinear energy operator (NEO). Finally, the extracted features of each frame are used to detect speaker change point which is accomplished by applying dissimilarity measures to find the distance between two frames. To realize it, a sliding window is moved across the whole data stream to find the highest peak which corresponds to the speaker change point. The distance metrics incorporated are standard “Bayesian Information Criteria (BIC)”, “Kullback Leibler Divergence (KLD)”, “T-test” and proposed algorithm to detect the speaker boundaries. At the end, threshold value is applied and their results are evaluated with Recall, Precision and F-measure. Best result of 99.34% is shown by proposed distance metric with pyknogram as compare to BIC, KLD and T-test algorithms.

## 1. Introduction

Nowadays segmentation process plays a significant role in various areas of speech processing, image processing [1] and multimedia content analysis [2]. It focuses on the partition of input signals into different parts according to their attributes. The preprocessing of audio stream using segmentation separates noise, silence, and speeches and can be applied for audio transcription, word count, and speaker diarization [3,4], speaker recognition, clustering, and indexing [5]. Moreover, it segments the audio stream into silence, speech, speaker, noise, music and other acoustic signals by detecting its boundaries [6]. A review article [7] published in 1998 provides a succinct review of speech research indicating its past, present, and future. To identify features of the speech signal for nonspeech/speech detection having low linguistic information is illustrated in [8]. The content analysis of audio for segmentation and classification, in which stream of an audio is segmented according to audio type or speaker identity is described in [9], A novel technique, DIS_T2_BIC, when no prior knowledge of speakers is assumed for audio speaker segmentation is represented in [10]. A new online method based on Bayesian information criterion (BIC) and the normalized cross-likelihood ratio (NCLR) is illustrated in [11]. The deep learning and audio segmentation research trends are described in [12] via Source-Wise Analysis. The three main categories of audio segmentation are metric-based, model based and hybrid method [13]. Metric based segmentation does not require training data and is used to calculate the distance between two segments containing speech signal. For same speakers the distance value is close to zero and highest value represents different speakers. Also, the point at which the distance value is highest corresponds to speaker change point. BIC [14,15], KLD [8,16] and generalized logliklihood ratio (GLR) [17,18] are the most commonly used distance metric algorithms. Model-based audio segmentation requires training data to train the speaker classes to form a set of models for classification. Combination of both techniques is known as hybrid algorithm in which pre-segmentation uses metric based algorithm followed by model based algorithms and improves the segmentation results [19]. Most of the algorithms performs well for speech segment length greater than 25 milliseconds but degrades its performance for short duration. A comprehensive overview of deep learning-based diarization and its challenges, is illustrated in [20].

### 1.1 Objectives

To overcome the limitations and problems of speaker segmentation system based on existing distance metrics, this study aims to develop a novel speaker change point detection system by accomplishing the following tasks:

1. Obtain standard multi-speaker audio recordings for the development and testing of the system;
2. Develop an algorithm that efficiently separates the speeches, non-speeches and overlapping speeches with music and further enhance the speech specific features;
3. Propose a new distance metric algorithm based on NCLR that successfully detects the speaker change points;
4. Evaluating proposed model performance by Recall, Precision and, F_Measure.

### 1.2 Contribution

The problem arises to the jail authorities when culprit talks to their family members on phone call in coded form of very short duration. The authority couldn’t be able to recognize the speech of third person in their recordings. The ability to estimate the speaker numbers and the speech information present in the multi-speaker audio recording over intervals is valuable in detecting culprit in forensic sciences. The main contribution of this paper is to the development of a novel system based on Pyknogram, discrete energy operator, DWT, and NCLR algorithms. Implementation of pyknogram and discrete energy operator extracts the unique features of speeches of the individual. These features are then used for speaker change point detection by applying proposed distance metric NCLR.

### 1.3 Structure of the paper

Firstly, this paper reviews the working and limitations of speaker segmentation process and then defines the objectives and contribution of the paper. The second section demonstrates the processing steps of speaker segmentation system that incorporates preprocessing, extracting features, change in speaker point detection, and evaluation criterion. Experimental results and system performance evaluation is discussed in third section. The final section concludes the research work.

## 2. Proposed speaker segmentation system

**Fig 1:**
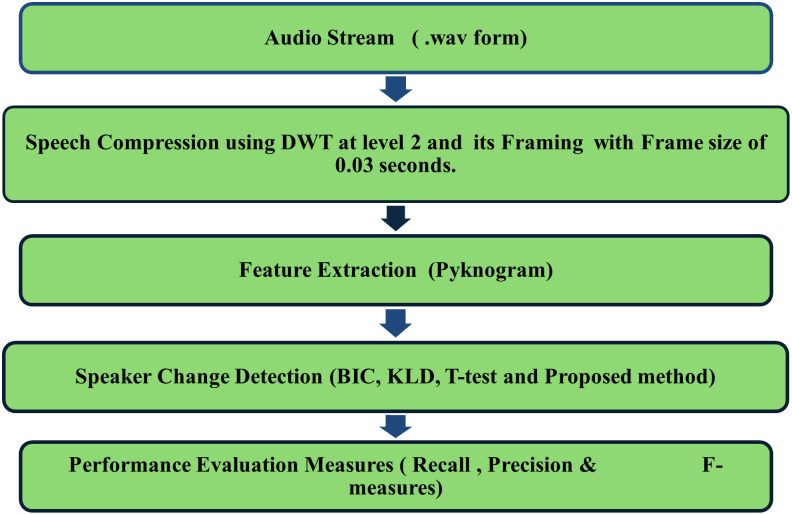
Proposed diagram of speaker segmentation system.

The aim of segmenting speaker is to segregates stream of audio into acoustically identical segments using efficient techniques. In this research work without using voice activity detection, the proposed system design is achieved by following basic steps and shown in Fig 1.

- Preprocessing and framing;
- Feature extraction using pyknogram;
- Speaker Change Point (SCP) detection based on proposed distance metrics, NCLR;
- System performance evaluation and comparison with other distance metrics.

### 2.1 Preprocessing using DWT and framing

The first step of speaker segmentation system is preprocessing of multi-speaker audio stream. The audio recording carries speeches of various speakers, noises, silent, clapping sound and music. The unwanted signals of silence noise, clapping sound and music must be removed and enhance the strength of speech signal. To do this, an efficient technique known as wavelet transform is implemented. Wavelet is a small wave which starts from zero, oscillates and then dimishes to zero. Wavelet transform is the enhanced form of Fourier transform which represents two parameters of time and Frequency on single graph. Due to the high frequency and time resolution property of DWT, it is applied to investigate a signal simultaneously in time-frequency domain and solves many technical issues in the field of Science, Mathematics, and Engineering. It can be used as signal compression technique, pattern recognition, Image or speech de-noising and scaling of the weak signal [21]. This paper implements DWT (Daubechies wavelet db40 at level 2) to decompose the speech signal into consecutive stages of high and low frequency coefficients [22]. The high frequency coefficients are known as details which carries noisy components of audio recording, and the low frequency coefficients known as approximations carries about 99% of speech information. Its mathematical expression is expressed by Eq (1) [21].

$${\text{W}}_{\psi }\text{x}\left(\text{m},\text{n}\right)=\frac{1}{\surd \text{m}}\int_{\infty }^{-\infty }\text{x}\left(\text{t}\right)\psi *\left(\frac{\text{t}-\text{n}}{\text{m}}\right)\text{dt},$$ (1)

Where, mother wavelet is:

$$\Psi_{\text{m},\text{n}}\left(\text{t}\right)=\Psi \left(\frac{\text{t}-\text{n}}{\text{m}}\right)$$ (2)

**Fig 2:**
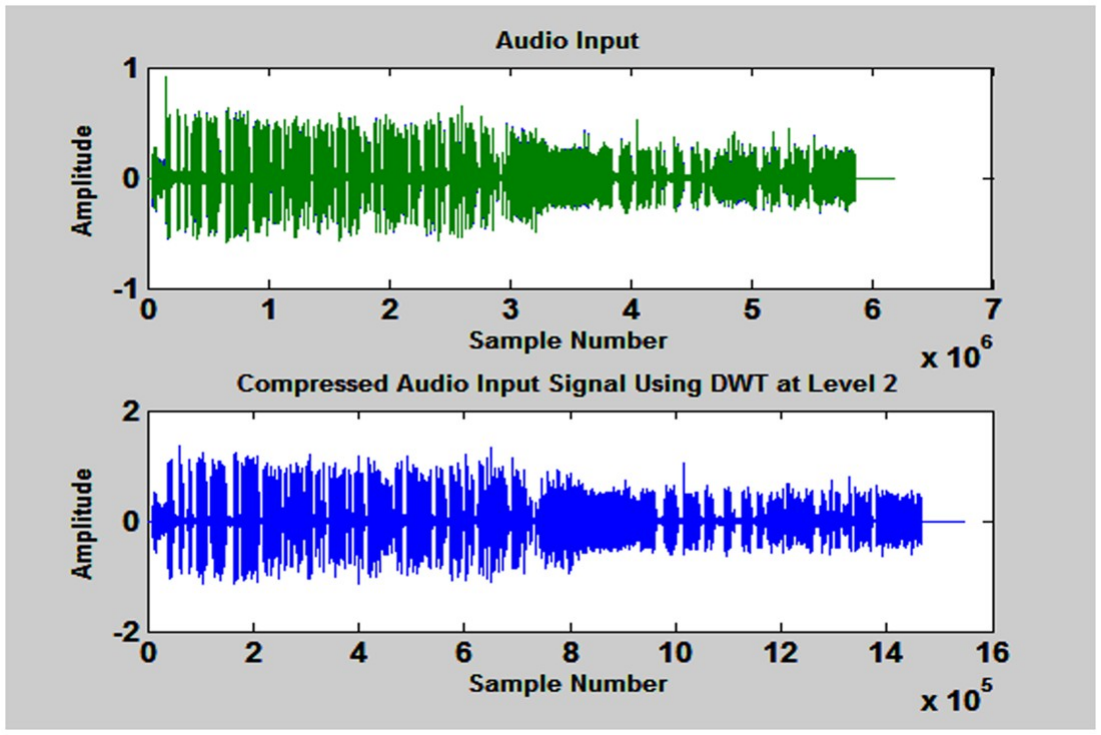
First figure represents the waveform of input signal and second shows its co-processed form in the ratio of 4:1 using DWT.

Where, m and n are scale and shift parameters respectively. The original audio stream and its corresponding scaled-compressed forms are depicted in Fig 2. It has two subplots; upper waveform shows original speech signal which has 6 X10^6^ Samples and lower subplot is its compressed form and has 15.5 X10^5^ samples known as approximation.

**Fig 3:**
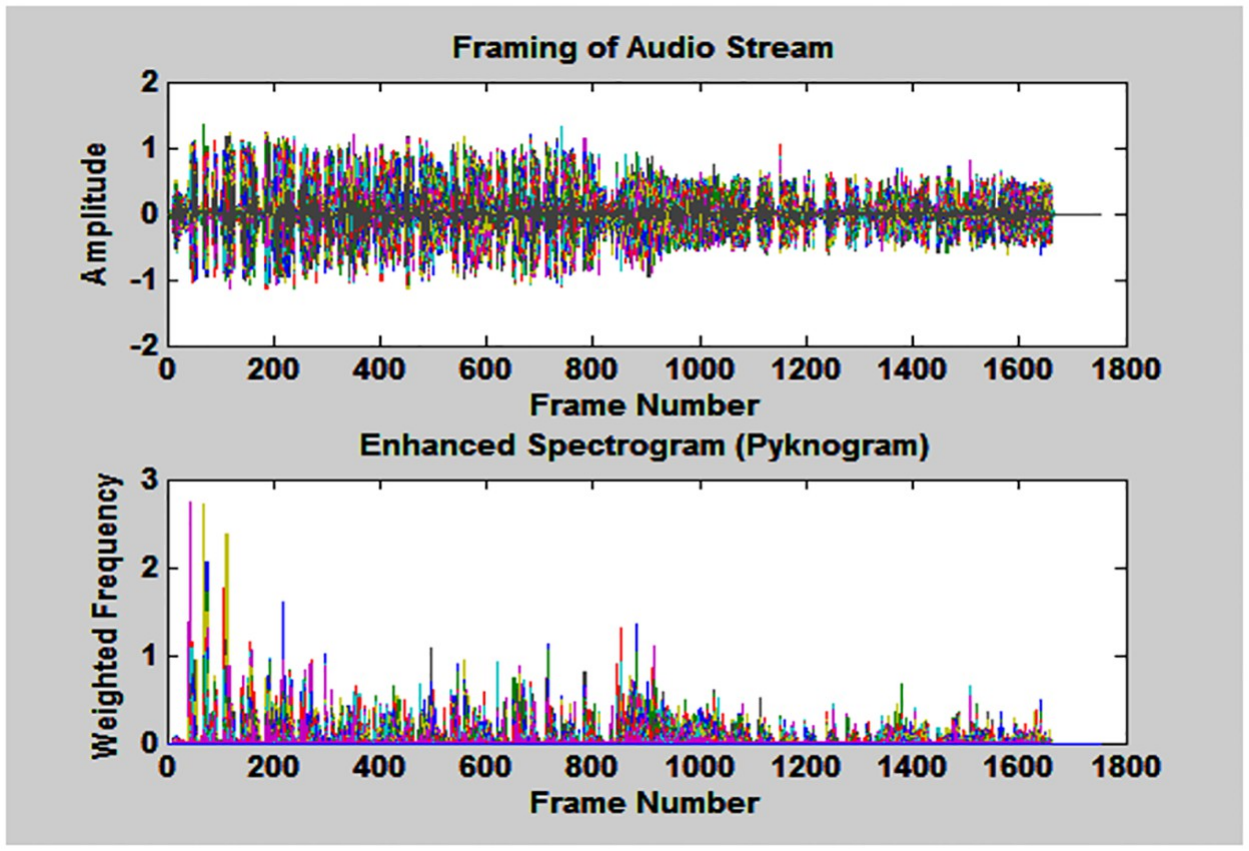
Upper subplot depicts frames of audio-stream and lower subplot represents extracted features of each frame.

As the length of the waveform is too large for further processing, so, it is converted into overlapping frames by applying hamming widow technique. Duration of each frame is 0.03 seconds (i.e., 1323 samples) with frame shift of 0.01 seconds (i.e., 441 samples) shown in upper subplot of Fig 3 [16].

### 2.2 Feature extraction using pyknogram

Pyknogram is an enhanced form of spectrogram of speech signal. Basically, spectrogram is a three-dimensional graph, illustrating the amplitude or signal energy over time at various frequencies. The vertical axis of the graph corresponds to frequencies, horizontal axis represents time, and the color of the graph illustrates amplitude or loudness of the signal. Dark blue corresponds to low amplitude and brighter colors up through red corresponding to progressively stronger amplitudes. This spectrogram was first enhanced to track the formant frequencies of audio signal and named as pyknogram [23] and is also used to detect the overlapping frames in speech data [24]. The basis of pyknogram is “Nonlinear Energy Operator” is given by the following Eq (3) [25],

$$\psi \left|x\left(n\right)\right|=x{\left(n\right)}^{2}-x\left(n-1\right)*x\left(n+1\right)$$ (3)

Where, ‘n’ represents the sample number of digitized speech.

In this research work, pyknogram is used to enhance the formant frequency expressed by Eq (4) and amplitude of the speech signal is reckoned by Eq (5).

$$f(n)=\frac{1}{\;2\pi }arccos(1-\left(\frac{\psi \left|x\left(n\right)-x(n-1\right|}{2\psi \left|x\left(n\right)\right|}\right)$$ (4)

$$\left|a\right|=\sqrt{\frac{\psi \left|x(n)\right|}{{sin}^{2}(2\pi f)}}$$ (5)

Where, f is frequency and |a| is amplitude of signal.

The dominant frequency of each frame of duration 0.12 seconds is computed by using Eqs (4) and (5) as follows:

$$F_{w}\left(t\right)=\frac{\sum_{t}^{n+T}f\left(n\right)a^{2\;}\left(n\right)}{\sum_{t}^{n+T}a^{2}\left(n\right)}$$ (6)

Where, a(n) and f(n) in Eq (6) are the amplitude functions and instantaneous frequency. These two are calculated for each sample in the t^th^ frame over the frame length (T samples per frame). Logarithm value of F_w_(t), is calculated and expressed in Eq (7).

$${\text{G}}_{\text{w}}\left(\text{t},\text{f}\right)=20\text{log}\left(\sum_{\text{t}}^{\text{n}+\text{T}}\text{f}\left(\text{n}\right){\text{a}}^{2\;}\left(\text{n}\right)\right)-20\text{log}\left(\sum_{\text{t}}^{\text{n}+\text{T}}{\text{a}}^{2}\left(\text{n}\right)\right)$$ (7)

Where, G_w_(t,f) is the logarithmic value of weighted average of instantaneous frequency components that enhances the output of pyknogram. The final time-frequency representation of G_w_(t,f) is depicted in the second subplot of Fig 3.

### 2.3 Distance metric for similarity measures

The third step of speaker segmentation system is speaker change point detection which has been accomplished by feature matching measures. As discussed earlier that audio recording was preprocessed and converted to frames of duration 0.12 seconds carrying 1323 samples per frame. Then pyknogram was applied on each frame to extract the features of speeches contained in the frames. To find the boundaries of speaker changes in the Multi-speaker audio recording, the four similarity measures were applied. These are Bayesian Information Criterion [26], Kullback-Leibler Divergence [27], T-test [28] and Proposed distance metric.

#### 2.3.1 Bayesian information criteria

To facilitate segmentation or speaker change point detection, the most commonly and efficient method applied is BIC or Schwarz Information criterion [13,29]. Its main function is to find the distance between two frames which is represented by ΔBIC and mathematically represented by Eq (8).

$$\Delta \text{BIC}=\left(\text{N}\right)\text{log}\left(\sum \right)-{\text{N}}_{1}\text{log}\left(\sum_{1}\right)-{\text{N}}_{2}\text{log}\left(\sum_{2}\right)-\lambda \left(0.5\left(\text{d}+0.5\left(\text{d}+1\right)\right)\right)\text{log}\left(\text{N}\right)$$ (8)

Where N_1_, N_2_ and N are the number of samples in frame1, frame2 and (frame1 + frame2) respectively and λ = 10. Similarly, ∑_1_, ∑_2_ and ∑ are the determinants of covariance matrices for frame1, frame2 and (frame1 + frame2) respectively, λ is a penalty weight, and d is a dimension of the feature space.

Since, the Multispeaker audio recordings have multiple frames, so, to find speaker change boundary, a sliding window is moved across all the frames to compute ΔBIC. If ΔBIC is greater than zero for two frames, then it represents similar frames otherwise that particular frame contains speaker change point and it belongs to different speaker. The main disadvantage of BIC is that it does not perform well on short duration data frames.

#### 2.3.2 Kullback-leibler divergence (KLD)

The KLD is a statistical method of computing distance between two populations, segments or frames [13]. In this paper, it has been applied for speaker segmentation to measure similarity between two frames. If N(μ_1_,∑_1_) and N(μ_2_,∑_2_) are the multivariate Gaussian distribution of two audio frames respectively, then their similarity can be evaluated by KLD distance metric expressed in Eq (9).


$$KL=\frac{1}{2}{{\left(\mu_{1-\;}\mu_{2}\right)}^{T}(\sum }_{1}^{-1}+\sum_{2}^{-1})\left(\mu_{1-}\mu_{2}\right)+\frac{1}{2}tr\left(\sum_{1}^{-1}\sum_{2}\sum_{2}^{-1}\sum_{1}-2I\right)$$ (9)

The highest value in a segment shows the dissimilarity and can be considered as speaker change point. This method gives better results for segment length greater than 5 seconds.

#### 2.3.3 T-Test

Speaker changes point detection using Student T-test is an efficient and extensively used technique for similarity measure in speech processing [17] and object based classification [30]. To check whether two speech frames belong to same speaker or to different speaker, a competent algorithm is required to find the distance between them. In this paper, T-test is applied on the frames which are usually next to each other, to detect the boundaries of the speaker changes. Mathematically, it is represented by Eq (10).

$$T_{d}=d\left(S_{1}\left(X\right),S_{2}\left(X\right)\right)=\frac{\left|m_{1}-m_{2}\right|}{\sqrt{\frac{\sigma_{1}^{2}}{n_{1}}}+\frac{\sigma_{2}^{2}}{n_{2}}}$$ (10)

Where S_1_(X) and S_2_(X) illustrate two frames with m1,σ1, n1, m2, σ2, n2are their respective mean, standard deviation and size. The distance between frames S1 and S_2_ is computed using Eq (10); a smaller value of T_d_ signifies those two frames belongs to the same speaker and its larger value indicates that the two frames belong to different speaker.

#### 2.3.4 Proposed distance metric algorithm

In this research work, to measure dissimilarity between two speakers, an efficient distance metric based on Normalized Cross Likelihood Ratio is proposed which was earlier used in speaker diarization system [11,27,31]. If features of two audio segments are A and B of length n and m respectively and size of feature space is p, then ∑_A_, ∑_B_ and ∑_AB_ represents covariance matrices determinants for the segments A, B and fused AB respectively. The proposed distance metric is defined in Eq (11).

$$Distance=\frac{\frac{log(\sum A)-log(\sum AB)}{n}-\frac{log(\sum B)-log(\sum AB)}{m}}{\lambda Q}$$ (11)

Where λ = 10, *Q* is the equalizing factor, and its value is:

$$Q=0.5\left(p+\frac{p\left(p+1\right)}{2}\right)\text{log}\left(n+m\right)$$ (12)

The two audio segments are similar for more positive distance value. Distance value closer to zero shows speaker change points. This algorithm gives better results for the detection of SCP in the speech duration of length 3–5 seconds.

### 2.4 Performance evaluation

Performance of speaker change point detection can be evaluated by Confusion matrix which is used to reckoned the precision, recall and F-measure [32,33]. For checking the Speaker Change Point (SCP), whether it belongs to the specified frame or not, the evaluation process results in four possible outcomes as shown in Table 1. hit (SCP is present and its predicted value is “Present’’), miss (SCP is present in the frame and the predicted value is “Absent’’), false alarm (SCP is absent in the frame and the predicted value is “Present"), and correct rejection (SCP is absent in the frame and the predicted value is “absent"). Two types of errors were detected in these four outputs: false alarms and missed detections.

- Missed detection (Error 1): Speaker is not attributed when the SCP exists in the frame.
- False alarms (Error 2): Speaker is attributed when there is no SCP in the frame.

**Table 1:** Confusion matrix: Describes the two errors based on ground truth and predicted values of the SCP in the frame.

Ground Truth		Existence of Speaker in the frame	
Prediction		Present	Absent
**Practical Decision for the existence of speaker in the frame**	**Present**	Hit or TP*(Correct decision)*	False Alarm or FP*(Error 2)*
	**Absent**	Missed Detection or FN*(Error 1)*	Correct Rejection or TN*(Correct decision)*
		P = TP +FN	N = FP +TN

Where, TP: True positive, TN: True Negative, FP: False positive, False Negative.

This table is used in computing recall, precision, and F-measure to investigate the performance of the speaker segmentation system.

Precision is the ratio of TP and (TP+FN) as expressed in Eq (13).


$$Precision=\frac{Number\;of\;correctly\;detected\;speaker\;changes(TP)}{Number\;of\;Speaker\;Changes\;detected\;in\;hypothesis(TP+FP)}$$ (13)

Recall is the ratio of TP points to all the speaker change points in reference file defined in Eq (14).


$$Recall=\frac{Number\;of\;correctly\;detected\;speaker\;changes(TP)}{Number\;of\;Speaker\;Changes\;in\;reference(TP+FN)}$$ (14)

The performance evaluation method “F-measure” which is the weighted average of Precision and Recall is reckoned in the following Eq (15).


$$F-Measure=\frac{2*Recall*Precision}{Recall+Precision}$$ (15)

Their values vary from 0 to 1. System performance is good for higher value of F-measure.

## 3. Results

### 3.1 Database used

The database uses the recordings of utterances of 11 speakers of 15–20 seconds were taken from the Personal Digital Assistant (PDA) speech database in .wav form [34]. These recordings are concatenated in a single recording and used as development data for speaker segmentation system. For the testing of proposed system, two recordings of TV news and TV show of duration 2–3 minutes were used at sampling frequency 44100Hz. TV news carries the recording of Last ten hours of Dr. A P J Abdul Kalam that includes background sad music, silence and speakers voices and the second recording of famous TV show entitled “Dr. Subhash Chandra Show” that carries background music, clapping, silence and speakers voices of short and long durations, act as test data source. Since, the recordings are in MP 3 format, to use it in MATLAB, it is converted into .wav form.

### 3.2 Experimental results

In this section, based on features extraction and speaker change point detection methods, experiments were performed on two different data sources development database and test database. It elaborates the effect of traditional distance metrics including, BIC, KLD, T-test and proposed algorithm, in detecting speaker boundaries where the speaker change occurs. Initially, development data source was processed and analyzed by speaker segmentation system and then it was tested by test data source.

**Fig 4:**
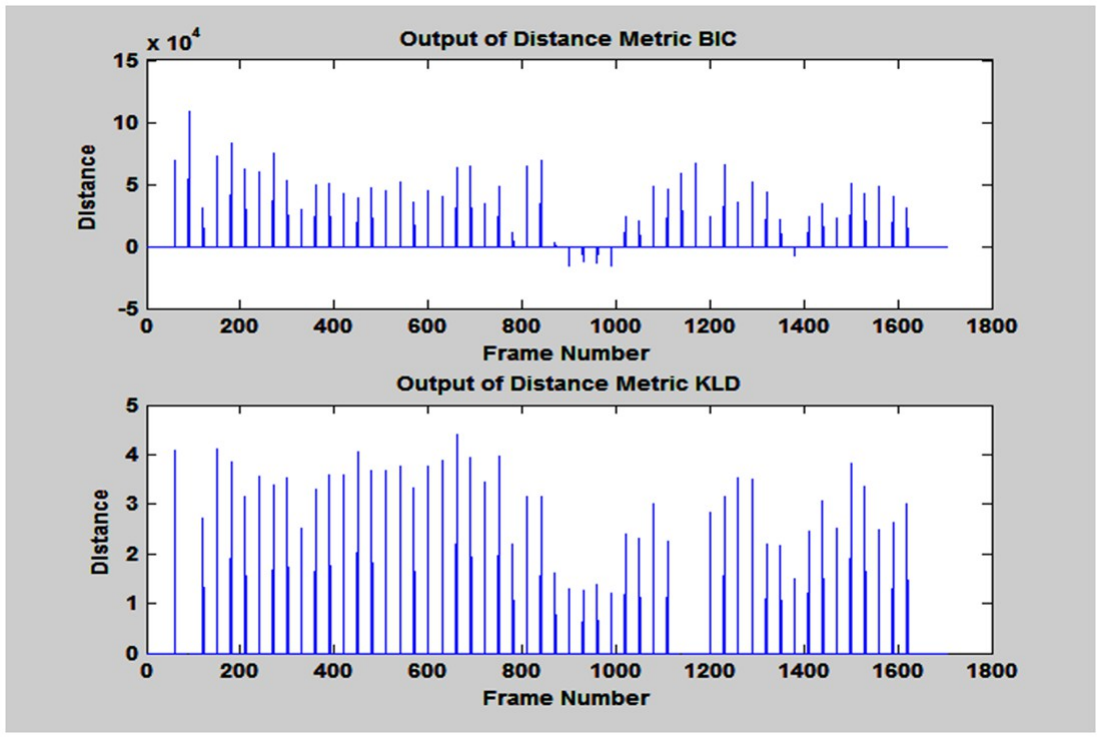
Outputs of BIC and KLD distance metrics.

In section 2, it was already discussed that the features of preprocessed speech signal were extracted by pyknogram, which is the logarithmic value of “weighted average of the instantaneous frequency components” depicted in Eq (7). In order to find the SCP, firstly, BIC was applied on frames for its feature matching. To accomplish this, a sliding window of 30 samples was moved across the whole audio stream to find the distance between two frames. The positive values of BIC or greater than threshold value simply that the frames are belonging to same speaker and vice-versa as shown in Fig 4.

At threshold level less than zero, it detected five change points out of which two were correct. When KLD distance metric was applied at threshold level 4, only nine boundaries were detected, out of which only three were correct. At threshold value 3.8, nine change points were detected which results in increase in false alarm rate and reduces its performance.

**Fig 5:**
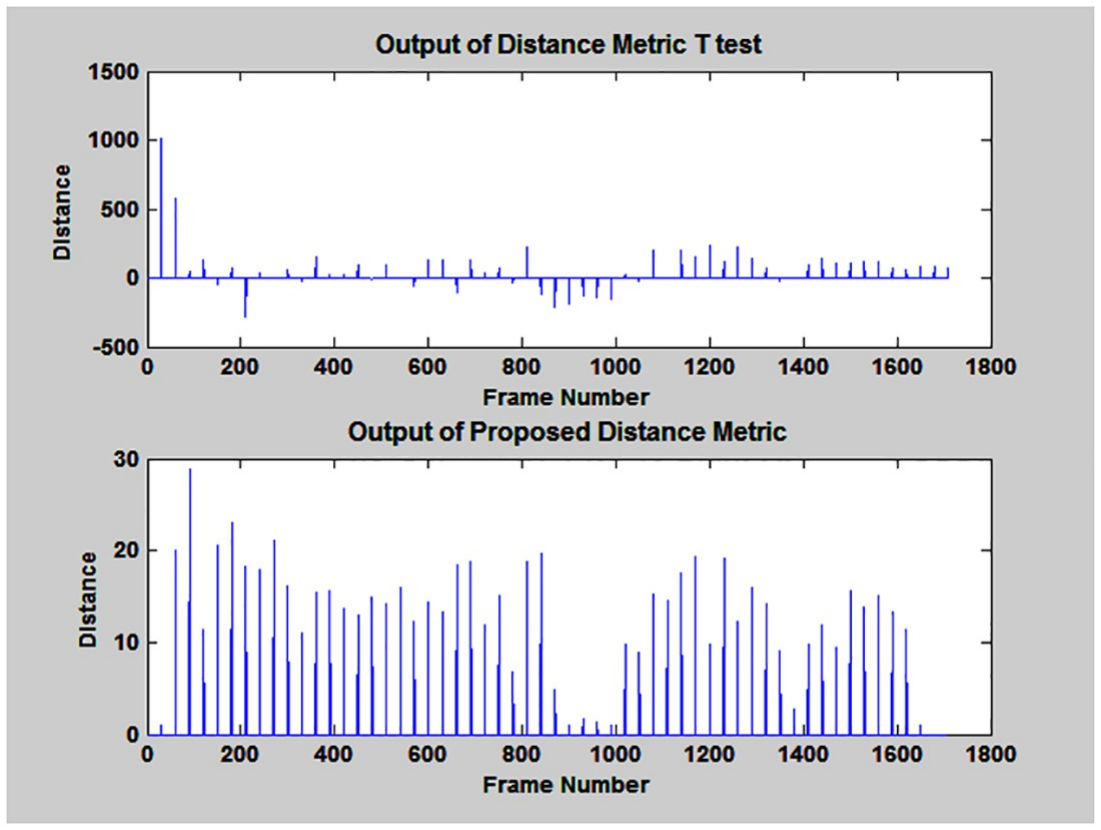
Outputs of T-test and proposed distance metrics.

Thirdly, T-test distance metric was implemented and their results are graphically represented in Fig 5. It shows that smaller distance value of its output reveals the same speaker. Moreover, at threshold level less than 70 and greater than zero, ten change points were detected. At last, proposed distance metric was applied and detected eight speaker change points corresponding to frame number 30, 860, 890, 910, 940, 1400 and 1630 at threshold value 2.

**Fig 6:**
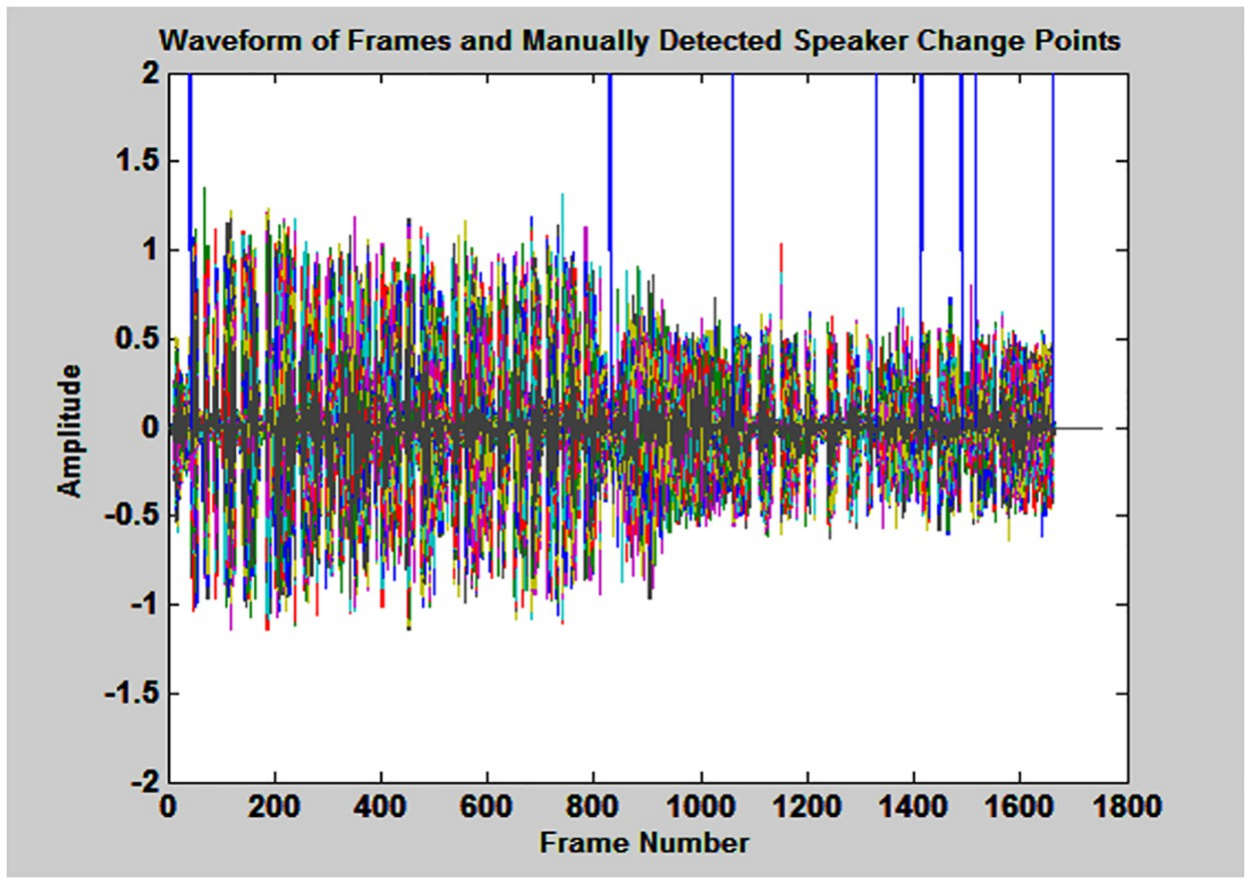
Manually detected 11 speaker change points and framed signal of development database.

Finally, their Performance was evaluated by recall, precision, and F_measure by using Eqs (13)–(15). These measures require ground truth of speaker segmentation, so, it was detected manually by using signal Processing tool (SPTOOL) in MATLAB and graphically represented in Fig 6. It contains two overlapping figures: manually detected speaker change points (Blue color) and frames of audio recording.

The frames of manually detected speaker change points were taken as reference points and are used to compare with hypothesized speaker change points for the computation of “Recall”, “Precision” and “F_measure”. The results of three existing distance metrics BIC, KLD, T-test and proposed distance metric are depicted in Table 2.

**Table 2:** Experimental results of speaker segmentation system for three existing distance metrics and proposed distance metrics applied on development dataset and test datasets.

Distance Metric	Threshold Value	“Recall” (%age)	“Precision” (%age)	“F-measure” (%age)
**Development Database (Actual Number of Speaker Change Points = 11)**				
**BIC**	Less than zero	95	63.1	75.83
**KLD**	Less than 3.8	99	42.13	59.10
**T-test**	Less than |70|	97.5	99.5	98.489
**Proposed Method**	Less than 2	100	98.7	99.34
**Test Database 1 (Actual Number of Speaker Change Points = 21)**				
**BIC**	Less than zero	91	53.3	67.22
**KLD**	Less than 3.8	100	22.22	36.36
**T-test**	Less than |70|	87.5	100	93.33
**Proposed Method**	Less than 2	100	88.89	94.12
**Test Database2 (Actual Number of Speaker Change Points = 21)**				
**BIC**	Less than 0	89	53.3	66.67
**KLD**	Less than 3.8	85	35	49.58
**T-test**	Less than |70|	86	98	91.61
**Proposed Method**	Less than 2	97	91	93.90

Furthermore, performance of speaker segmentation was also tested by test database and evaluated by following the same steps as already discussed. When BIC was used, 10 change points were detected at threshold value 3.8. Results from KLD distance metric reveals less favorable as compared to BIC because it creates more false alarms. T-test and proposed distance metric again shows comparable results at threshold value 70 and 2 respectively.

### 3.3 Discussion

The performance results of all the algorithms were tabulated in Table 2. The proposed distance metric with pyknogram at threshold value 2, gives improved results of 99.34% 94.12% and 93.90% for development database, Database 1 and Database 2 respectively, when compared to the existing distance metrics BIC, KLD and T-test. Evaluation results shows that proposed method lucidly separates the speakers and scores highest value of F_measure which is very close to T-test distance metric. Also, there is loud clapping sound in the beginning and end of speech which is also clearly detected. When it is compared with the results of manually segmented frames, speech segments and clapping sound of short duration (less than 5 seconds) were not correctly detected. It can be improved by increasing the length of sliding window and frame size.

**Fig 7:**
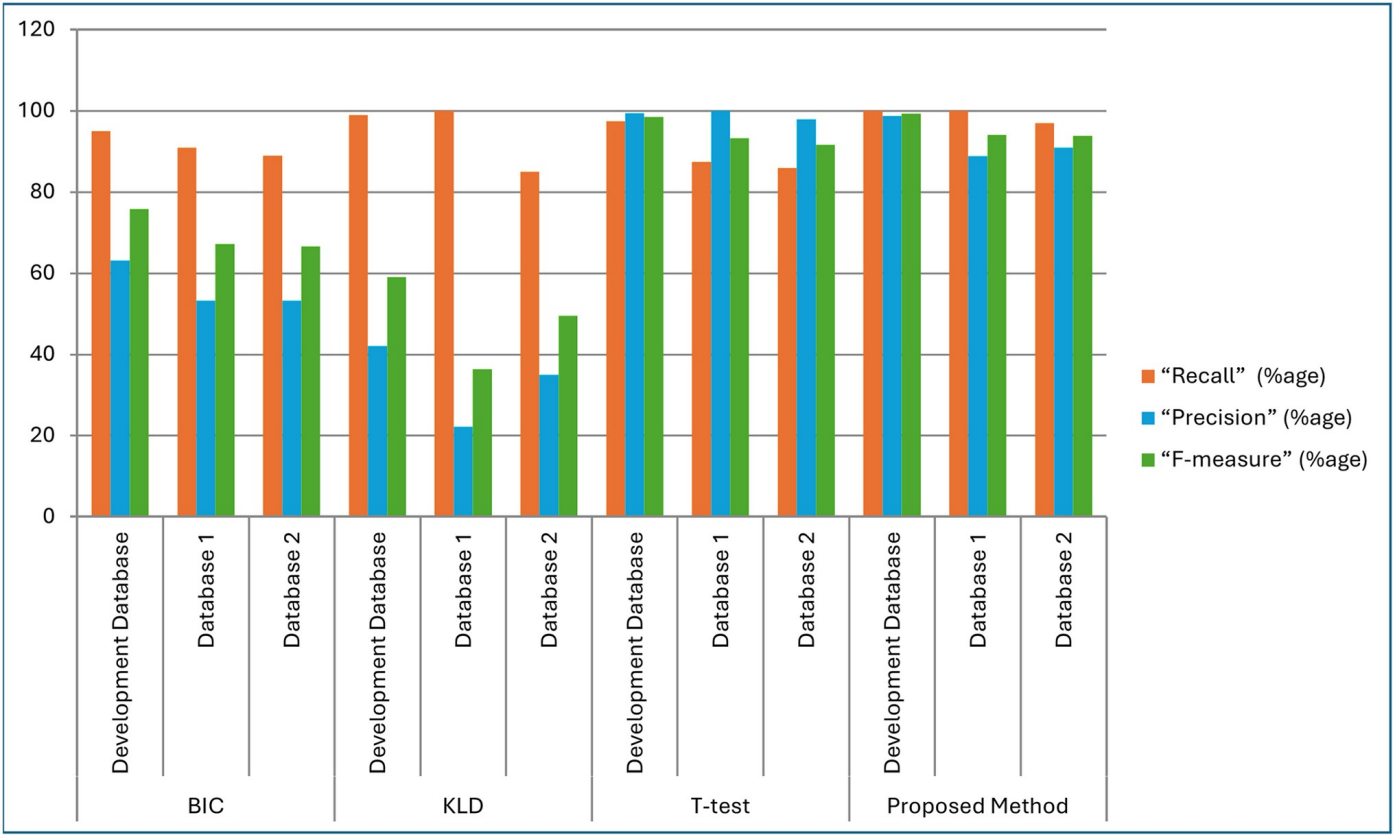
Performance of proposed method for speaker change point detection and traditional distance metrics with various databases.

Fig 7 illustrates the graphical representation of results obtained after analyzing and evaluating the proposed distance metrics and existing distance metrics BIC, KLD and T-test for three databases. It shows that when speech length is less than 5 seconds as in the case of database-1 and database-2, some segments were not detected properly and results lower F-Measure. But in Development database, the speech lengths are greater than 5 seconds and maximum speaker change points were detected which results in higher F-measure. Overall result of the proposed system shows better F_measure than the traditional methods [35,36].

### 3.4 Findings and their significance

The aim of this research is to propose an efficient speaker change point detection model that uses discrete energy operator based pyknogram and proposed distance metric algorithm, NCLR. This method successfully enhances the weak speech signal, suppresses its noise and measures the variability of the spectrum over the time for different speakers. Implementation of the proposed technique has given better results of F-measure in the process of speaker segmentation than the existing techniques of distance metrics, BIC, KLD and T-test as shown in Table 2. The scope of this proposed method is in Forensic speaker recognition applications where the speaker’s voice is first detected in a multispeaker audio recording and then segmented to extract the information carried in it. This method works well for speeches of duration greater than 5 seconds but to extract the speech information from speeches of duration less than 3 seconds is very challenging in an application of speaker diarization.

## 4. Conclusions

In speech processing, segmentation and clustering algorithms plays a vital role in speaker recognition, speaker diarization, word count, and audio transcription. In this research paper, a novel distance metric algorithm has been proposed to find the boundaries at which speaker changes in the recording of audio conferences. Basically, in this research work, two databases: development data and test data were used. Initially, the audio recording of development data was compressed and denoised, at threshold value 0.03, using discrete wavelet transform and then it was partitioned into frames. The features of each frame were extracted by pyknogram in which logarithmic value of weighted average of instantaneous frequency was calculated. Then, distances between frames were obtained by applying distance metrics: BIC, KLD, T-test and proposed algorithm, with the help of sliding window, to detect the boundaries of speakers. Furthermore, by following the same processing steps of segmentation, test datasets were applied and their results were evaluated by using F-measure. It is concluded that the proposed distance metric with pyknogram at threshold value 2, gives improved results of 99.34% 94.12% and 93.90% for development dataset and two test datasets respectively when compare to other distance metrics. In future, the proposed technique could be enhanced to handle the speeches of short duration (< 3 seconds) in speech processing.
