# Correction: Optimized technique for speaker changes detection in multispeaker audio recording using pyknogram and efficient distance metric

**DOI:** 10.1371/journal.pone.0320922

**Published:** 2025-03-25

**Authors:** The PLOS One Staff

The first and sixth authors, Sukhvinder Kaur and Punit Gupta, should not have been attributed equal contribution to this work.

The first and seventh authors, Sukhvinder Kaur and Ali Nauman, should be noted as contributing equally to this work.

The publisher apologizes for the error.
